# Scorpion-Centipede extracts mitigate ovariectomy-induced osteoporosis in mice through facilitating Cx3cr1 expression

**DOI:** 10.3389/fphar.2025.1604096

**Published:** 2025-10-24

**Authors:** Jinghuai Ni, Lingling Yu, Bingjie Wang, Shuai Chen, Wenbin Shang, Penghua Fang, Bin Du, Wen Min

**Affiliations:** ^1^ Department of Bone Injury of Traditional Chinese Medicine, Affiliated Hospital of Nanjing University of Chinese Medicine, Nanjing, China; ^2^ Key Laboratory for Metabolic Diseases in Chinese Medicine, First College of Clinical Medicine, Nanjing University of Chinese Medicine, Nanjing, China

**Keywords:** Scorpion-Centipede, Cx3cr1, osteogenic differentiation, adipogenic differentiation, PMOP

## Abstract

**Objectives:**

Scorpion and Centipede (SC) is an ancient formula of traditional Chinese medicine that is commonly utilized in a range of disorders, and it has been shown to have pharmacological effects on postmenopausal osteoporosis (PMOP). However, the specific mechanism of SC for the treatment of PMOP remains to be further investigated. This study aimed to investigate the therapeutic potential of a traditional Chinese medicine formula consisting of SC in regulating the osteogenic and adipogenic differentiation of bone marrow mesenchymal stem cells (BMMSCs) to treat PMOP.

**Methods:**

The ovariectomy-induced mice (OVX) were established and divided into a sham surgery group, OVX, OVX with alendronate sodium, and OVX with SC, and kept for 10 weeks. *In vitro* experiments were conducted to evaluate the effects of SC on osteogenic and adipogenic differentiation in BMMSCs, MC3T3-E1, and 3T3-L1 cells.

**Results:**

The results showed that SC treatment significantly improved bone mineral density (BMD), trabecular separation (Tb.Sp), trabecular thickness (Tb.Th), trabecular number (Tb.N), bone volume fraction (BV/TV), and trabecular pattern factor (Tb.Pf) in OVX mice. In addition, SC treatment markedly increased Runx2, Osx, Alp, and Cx3cr1 while decreasing adipogenic genes like PPARγ and C/EBPα in the bone tissue of OVX mice and BMMSCs. Notably, the effects of SC on osteogenic and adipogenic genes were blocked in Cx3cr1 knockdown MC3T3-E1 and 3T3-L1 cells.

**Conclusion:**

This study demonstrates that the SC effectively increases bone mass and osteogenesis by promoting Cx3cr1, thereby increasing osteogenic differentiation and inhibiting adipogenic differentiation. These findings amplified the mechanisms of SC and its potential to treat PMOP.

## Graphical Abstract

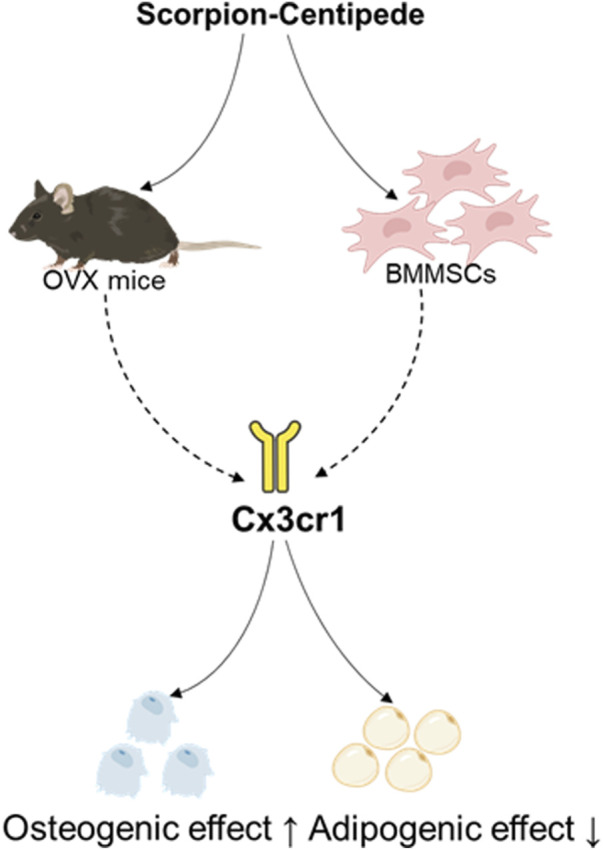

## 1 Introduction

Postmenopausal osteoporosis (PMOP) is a common bone disease in women, characterized by a sharp decline in estrogen levels leading to decreased bone density and bone fragility, significantly increasing the risk of fractures (Brown, 2021). Currently, the prevalence of osteoporosis among people aged 50 and above in China is 19.2%, with a higher rate of 32.1% for women; for those aged 65 and above, the prevalence is 32.0%, with an even higher rate of 51.6% for women (Zeng et al., 2019). Therefore, PMOP has become a serious public health issue that urgently needs to be addressed. In clinical practice, the most common medications used to treat PMOP are either anti-resorptive or bone-forming medicines, such as bisphosphonates, estrogen medications, and calcitonin. On the other hand, prolonged use of these medications may result in a variety of negative consequences on the cardiovascular system, breasts, and prostate (Appelman-Dijkstra and Papapoulos, 2015). Long-term use of bisphosphonates can lead to severe complications, such as osteonecrosis of the jaw (Park et al., 2025) and atypical fractures (Murphy et al., 2022). Therefore, the search for affordable, low-toxicity, and efficient alternative treatments for PMOP is crucial.

The primary cause of PMOP is an imbalance between bone resorption and creation. PMOP develops when bone resorption outpaces bone production, causing the amount of bone to continue decreasing (Eastell and Szulc, 2017). Therefore, the principal objective of PMOP treatment will be to control the ratio of bone production to resorption. The synthesis, secretion, and mineralization of bone matrix are carried out by osteoblasts, which are the main functional cells involved in bone formation. Bone marrow mesenchymal stem cells (BMMSCs) are the primary source of osteoblasts. The lineages into which BMMSCs can differentiate include adipocytes, chondrocytes, and osteoblasts. BMMSCs in bone marrow preferentially develop into adipocytes and osteoblasts, which are mutually exclusive (Chen et al., 2016). One major cause of decreased bone production is an imbalance in BMMSC differentiation, where adipocytes rose and osteoblasts decreased. Consequently, controlling the osteogenic-adipogenic balance of BMMSCs will be essential for PMOP therapy.

The scorpion-centipede (SC) is a traditional Chinese herbal medicine pair that is used to treat a variety of diseases in traditional medicine, such as arthritis (Liu et al., 2012), epilepsy (Rong et al., 2021), asthma (Zhang et al., 2023), and tumors (Hu et al., 2023). SC is a medication that is frequently used in traditional Chinese medicine to treat PMOP (Hui and Songnian, 2019; Weiming et al., 2019; Li, 2021; He et al., 2023; Jian et al., 2023; Jie et al., 2024). According to clinical research, people with postmenopausal osteoporosis (PMOP) can successfully reduce their feelings of bone pain by taking decoctions that contain the major constituents of SC or by adding SC to current therapies. Furthermore, in PMOP patients, they have the effect of enhancing bone metabolism and boosting bone density (Min et al., 2010). According to a preliminary study, SC can effectively boost the expression of the β-catenin protein in bone tissue, which would raise the rats’ bone mass and mineral density (Min et al., 2018). Furthermore, SC may efficiently suppress zebrafish autophagy gene expression, which lowers osteoclast differentiation and prevents bone resorption (Tan et al., 2020). Consequently, SC can enhance bone metabolism, decrease bone resorption, encourage bone formation, and more successfully prevent and cure PMOP. Additionally, studies have shown that the bone mineral density of OVX rats may be effectively increased by scorpion venom (Gomes et al., 2009). Although the SC has a clear therapeutic benefit in treating PMOP and includes many active components, its precise mechanism of action has not yet been determined. Therefore, the aim of this investigation is to first determine whether SC could be effective in treating PMOP by preventing BMMSCs from differentiating into osteogenic and adipogenic cells. Specifically, this study will evaluate the therapeutic effect of SC on PMOP based on changes in bone mass and bone quality of OVX mice after modeling. Additionally, by assessing the intervention effect of SC on BMMSCs, this study will verify the regulatory capacity of SC on osteogenic genes (Runx2, Osx, Alp) and adipogenic genes (PPARγ, C/EBPα) respectively. In the end, we will pinpoint the precise signaling pathways that SC regulate the osteogenic and adipogenic development of BMMSCs using transcriptomic approaches and offer comprehensive proof of the molecular mechanism of SC in the treatment of PMOP.

## 2 Materials and methods

### 2.1 Drugs and reagents

The scorpions and centipedes (see Supplementary Table S1) were purchased from Jiangsu Provincial Hospital of Traditional Chinese Medicine and were macroscopically and microscopically identified according to the Pharmacopoeia of the People’s Republic of China (2020 Edition). A voucher specimen of each herbal material was deposited in the Herbarium of the First Clinical Medicine Department Lab of Nanjing University of Traditional Chinese Medicine. Alendronate sodium was purchased from Beijing Zhendong Kangyuan Pharmaceutical Co., Ltd. with batch number 20190357. Runx2 (20700-1-AP), PPARγ (16643-1-AP), Ccr5 (82942-1-RR), Lst1 (21361-1-AP), Stra6l (22001-1-AP), β-Tubulin (80713-1-RR), and anti-rabbit IgG (H + L) (RGAR001) were purchased from Proteintech (Wuhan, China). Alp (A0514), C/EBPα (A25033), β-Actin (AC028), Gstm1 (A17492), Cx3cr1 (A2890), Pde4b (A23257), HK3 (A8428), Spn (A6412), Anpep (A23653), Gpnmb (A14270), Gmfg (A12955), Oas2 (A17428), and Marco (A10048) were purchased from Abclonal (Wuhan, China); Osterix (YN0332), Sel1l3 (YN5187), and Tnip3 (YN0333) were purchased from Immunoway; Slc39a4 (E1A19630C-1) and Rhov (E1A14851C-1) were purchased from Enogene (Nanjing, China); Hifair Ⅲ first Strand cDNA Synthesis SuperMix for qPCR (gDNA digester plus) (11141ES60) and Hieff qPCR SYBR Green Master Mix (Low Rox Plus) (11202ES08) were purchased from Yeasen (Shanghai). β-Actin (4970) and (14708) were also obtained. DMEM medium (high glucose) (6124078), αMEM medium (6124118), FBS (26010074), and NBCS (16010159) were purchased from Gibco (Waltham, United States); BCIP/NBT alkaline phosphatase color development kit (3206) and RIPA lysis buffer (BYTP0013B) were purchased from Beyotime (Shanghai, China). Mouse bone marrow mesenchymal stem cell osteogenic differentiation kit (MUXMX-90021), mouse bone marrow mesenchymal stem cell adipogenic differentiation kit (MUXMX-90031), mouse MC3T3-E1 cell osteogenic differentiation kit (MUXMT-90021), mouse 3T3-L1 cell adipogenic differentiation kit (MUXTL-90031), Alizarin Red S solution (ALIR-10001), and Oil Red O staining solution (OILR-10001) were purchased from Oricell (Guangzhou, China). Trizol reagent (YFXM0011P) was purchased from Yifeixue (Nanjing, China). The bone alkaline phosphatase (BALP) ELISA kit (product number: AF2775-A) and the tartrate-resistant acid phosphatase 5b (TRACP-5b) ELISA kit (product number: AF9392-A) were both purchased from Aifang Biotechnology (Hunan, China).

### 2.2 Drug preparation

The aqueous extract of SC is composed of equal parts (50% each) of scorpion and centipede. For each group, the herbal mixture was weighed at 10 times the prescription dose (approximately 100 g). The weighed herbs were crushed and decocted with water twice: the first decoction was performed by adding 10 times the volume of water and refluxing for 2 h, and the second decoction was carried out by adding 8 times the volume of water and refluxing for 1.5 h. The two extracts were combined, and the solvent was recovered under reduced pressure. The SC aqueous extract was concentrated to a concentration of 1 g crude drug per mL and stored in a refrigerator at 4 °C for subsequent use. An appropriate amount of the SC aqueous extract was placed in a 50 mL centrifuge tube, ensuring that the liquid volume did not exceed 1/3 of the centrifuge tube’s capacity. The tube was stored at −80 °C overnight. After the liquid was completely frozen, it was transferred to a freeze dryer for lyophilization until all moisture in the drug was removed. Subsequently, the lyophilized block-shaped Chinese medicine was crushed and pulverized to obtain a uniformly textured powdered freeze-dried Chinese medicine. It was determined that 100 g of the original SC crude drug yielded 16.4 g of freeze-dried powder (see Supplementary Figure S1). The obtained freeze-dried powder was identified by SCI-GO (Sci-go, Beijing, China).

### 2.3 Component analysis of scorpions and centipedes

The major chemical component analysis of SC: A portion of SC was processed into lyophilized powder. Accurately weigh 40 mg of the lyophilized sample, add 400 μL of 75% aqueous methanol, and perform ultrasonication for 30 min at 20 °C. Subsequently, centrifuge the mixture at 17,000 g for 10 min and transfer the supernatant to an injection vial for instrumental analysis. Following the mobile phase parameters outlined in Supplementary Table S2, detection was carried out using an AB 5600 Triple TOF mass spectrometer. The injection volume was 4 μL. SC was subjected to both positive and negative ion scanning modes, and primary and secondary mass spectrometry data were acquired through the IDA function. During each data acquisition cycle, molecular ions with the highest intensity (threshold >100) were selected for matched secondary MS data collection. Raw data were initially converted to abf format using AnalysisBaseFileConverter. The converted ABF files were processed with MSDIAL ver 4.6 software for peak detection, peak alignment, and other data processing steps. Compound identification was achieved by cross-referencing primary and secondary mass spectra against an integrated database combining Metlin, MassBank, MoNA, and HMDB. The results are presented in Supplementary Figure S2 and Supplementary Table S3.

To improve the pre-formulation study, this research supplemented Differential Scanning Calorimetry (DSC) and Fourier-Transform Infrared (FT-IR) analyses of scorpion and centipede extracts. DSC clarified the thermal behavior characteristics, providing a basis for formulation development and storage optimization; FT-IR presented information on functional groups, aiding in explaining the material basis of bioactive components (see Supplementary Figure S3). These supplementary data have enhanced the rigor of the study and the reliability of the results.

### 2.4 Animals and treatments

Sixty SPF-grade female C57BL/6 mice, aged 6 weeks and weighing 20 ± 5 g, were purchased from Qinglongshan Animal Breeding Farm in Nanjing, Jiangsu Province. The animals were housed at the Animal Experiment Center of Nanjing University of Chinese Medicine under controlled conditions with a temperature of 23 °C ± 2 °C, a relative humidity of 50% ± 15%, and free access to food and water. The animal experiment was approved by the Animal Ethics Committee of Nanjing University of Chinese Medicine (No. 202302A070). After a 2-week acclimatization period, bilateral ovariectomy was performed on the mice under anesthesia delivered via a gas anesthesia machine, with isoflurane as the anesthetic agent (see Supplementary Figure S4). Twelve weeks post-surgery, five mice from each group were randomly selected for euthanasia, and their right femurs were harvested. The muscles and connective tissues attached to the femurs were removed, followed by CT scanning to verify the success of the modeling. After confirming successful modeling, the mice were randomly divided into four groups: the SC group (scorpion and centipede), the positive control group AS (alendronate sodium), and the model control group OVX, with a sham-operated group Sham serving as a comparison. The SC group received gavage at a dose of 1.3 g/kg, while the AS group received gavage at a dose of 50 μg/kg body weight. Other groups were given 10 mL/kg normal saline by gavage per day, once daily, for 10 consecutive weeks. The dose for the treatment in mice was converted in reference to the traditionally used clinical dose and the commonly applied practice guide for the conversion of doses between humans and rodents (Reagan-Shaw et al., 2008). After the treatment period, blood was collected from the ocular globe, and sera, bone tissue, and adipose tissue were separated and stored at −80 °C. Additionally, three femurs and lumbar spines from each group were randomly selected and fixed in 4% paraformaldehyde.

### 2.5 Micro-computed tomography (micro-CT)

After 12 weeks of treatment, the mice were euthanized by cervical dislocation, and their femoral specimens were analyzed using micro-CT (Skyscan, Aartselaar, Belgium). The Skyscan 1176 micro-CT was set with a scanning spatial resolution of 9 μm. Following scanning, three-dimensional image reconstruction was performed using the N-Recon software integrated with the micro-CT. The CTAN software was utilized for three-dimensional analysis of the trabecular bone in the region of interest, yielding the following parameters: bone volume/total volume (BV/TV), trabecular thickness (Tb⋅Th), trabecular number (Tb⋅N), trabecular separation (Tb. sp), trabecular number (Tb.N), bone volume fraction (BV/TV), trabecular pattern factor (Tb.Pf), and bone mineral density (BMD).

### 2.6 ELISA

Detection of BALP and TRAP in mouse serum was performed using a mouse enzyme-linked immunosorbent assay (ELISA) kit according to the manufacturer’s instructions. Briefly, 10 μL of sample was taken for each assay point, mixed with 40 μL of sample diluent, and the contents of BALP and TRAP were analyzed by enzyme-linked immunosorbent assay. According to the manufacturer’s specifications, the intra-assay and inter-assay precision coefficients of variation (CV%) for both are <15%. All detections were performed in duplicate, and the final result was taken as the average of the two measurements.

### 2.7 Haematoxylin and eosin (H&E) staining

The femoral tissues of the mice were isolated and thoroughly fixed in 4% paraformaldehyde for 24 h. Subsequently, the tissues underwent decalcification in EDTA decalcifying solution for 1 month. After decalcification, the tissues were subjected to dehydration, wax immersion, and embedding to prepare paraffin sections. The sections were then dewaxed, stained with haematoxylin and eosin, dehydrated, cleared, mounted, and imaged under a microscope.

### 2.8 Cell viability

The CCK8 (Beyotime, Shanghai) assay was used to determine the effects of SC on the viability of BMMSCs, MC3T3-E1, and 3T3-L1 cells. After being seeded in 96-well plates, the cells were treated for 24 or 48 h with SC. After adding the CCK-8 solution, cells were incubated for 2 h, and the absorbance at 450 nm was measured.

### 2.9 Cell culture and treatment

Extraction and Isolation of BMMSCs: Five 6-week-old SPF-grade C57BL/6 female mice were euthanized by cervical dislocation after anesthesia. Following disinfection with 75% ethanol, the mice were placed on a sterile operating table, and both femurs and tibias were removed to collect bone marrow fluid. After centrifugation, BMMSC-specific medium was added. The medium was changed 24 h after extraction and subsequently every 2 days until the cells reached confluence. The second-generation cells were collected for cellular experiments.

The mouse osteoblast-like cell line MC3T3-E1 (Oricell, Guangzhou, China) was cultured in α-minimum essential medium supplemented with 10% fetal bovine serum (FBS) and 1% penicillin-streptomycin. The mouse adipocyte cell line 3T3-L1 (cell bank of the Institute of Biochemistry and Cell Biology, Shanghai, China) was cultured in Dulbecco’s Modified Eagle Medium (DMEM) containing 10% newborn calf serum (NBCS) and 1% penicillin-streptomycin. Both cell lines were maintained at 37 °C in a 5% CO_2_ atmosphere.

BMMSCs and MC3T3-E1 cells were subjected to osteogenic induction for 7 days, while BMMSCs and 3T3-L1 cells underwent adipogenic induction for 7 days. Following this, the cells were treated with an aqueous extract of scorpion and centipede for 24 h. The administered dosages were all determined based on the results of CCK8 experiments (Supplementary Figure S1). Subsequently, proteins or RNA were extracted for further experiments.

### 2.10 Transcriptome sequencing

Total RNA was extracted from rBMMSCs using TRIzol reagent. Transcriptome sequencing and bioinformatics analysis were conducted at Tiangen Biochemical Technology (Beijing) Co., Ltd. Purified mRNA was used for the preparation of cDNA libraries.

### 2.11 Western blot analysis

BMMSCs, MC3T3-E1, and 3T3-L1 cells were cultured in osteogenic or adipogenic induction media with or without different drug treatments for 7 days. The cells were then homogenized in RIPA lysis buffer containing 1% protease inhibitor and 1% phosphatase inhibitor. The total protein content of the cell lysates was quantified using a BCA Protein Assay Kit (Beyotime Biotechnology, China). Equal amounts of protein (60 μg) from each sample were separated by 8.5% SDS-polyacrylamide gel electrophoresis (SDS-PAGE) and transferred onto polyvinylidene fluoride (PVDF) membranes. The membranes were probed with primary antibodies against β-catenin, β-tubulin, and HRP-affinity purified goat anti-rabbit IgG. Proteins were detected using an enhanced chemiluminescence (ECL) detection reagent (Bio-Rad, United States) and visualized using a Bio-Rad imaging system with Quantity One analysis software.

### 2.12 RT-PCR analysis

RNA was extracted using Total RNA Extraction Reagent (RNApure) and then reverse transcribed into cDNA using Hifair Ⅲ first Strand cDNA Synthesis SuperMix for qPCR. Quantitative real-time PCR (qRT-PCR) was performed using the ABI-7300 TM Real-Time System (Applied Biosystems, United States). The 2^−ΔΔCT^ method was employed for calculation. The primer sequences are listed in Table 1.

**TABLE 1:** The oligonucleotide primers.

Gene	The oligonucleotide primers
GAPDH	Forward: GGT​TGT​CTC​CTG​CGA​CTT​CA
GAPDH	Reverse: TGG​TCC​AGG​GTT​TCT​TAC​TCC
Runx2	Forward: CTG​TGG​TTA​CCG​TCA​TGG​CC
Runx2	Reverse: GGA​GCT​CGG​CGG​AGT​AGT​TC
Osx	Forward: CTG​ACC​TTT​CAG​CCC​CCA​AA
Osx	Reverse: TGA​GGG​AAG​GGT​GGG​TAG​TC
ALP	Forward: CAT​AGT​CAC​GGC​CAG​TCC​TC
ALP	Reverse: ACC​CCG​CTA​TTC​CAA​ACA​GG
PPARγ	Forward: ATT​GAG​TGC​CGA​GTC​TGT​GG
PPARγ	Reverse: GGC​ATT​GTG​AGA​CAT​CCC​CA
C/EBPα	Forward: CGG​TGG​ACA​AGA​ACA​GCA​AC′
C/EBPα	Reverse: ACG​TTG​CGT​TGT​TTG​GCT​TT
Cx3cr1	Forward: GGG​TTT​GGT​GAG​TCC​TGG​TT
Cx3cr1	Reverse: CAA​GGA​ATG​GAC​ACC​CGA​CA
Ccr5	Forward: ATC​AGG​GCC​GGG​AAA​TAT​GC
Ccr5	Reverse: AGT​GGT​TCT​TCC​CTG​TTG​GC
Gstm1	Forward: ATG​GTT​TGC​AGG​GGA​CAA​GG
Gstm1	Reverse: TAG​TGA​GTG​CCC​GTG​TAG​CA
Pde4b	Forward: GCA​GAG​CCC​AGT​GAC​AAA​CT
Pde4b	Reverse: GCA​GAG​CCC​AGT​GAC​AAA​CT
Hk3	Forward: GCT​TGT​CGC​CTT​ACC​CAG​AT
Hk3	Reverse: GCA​GAC​GGC​TAT​AAG​GGG​AC
Spn	Forward: CTG​GTG​CCC​ATG​CTT​ATT​GC
Spn	Reverse: TTC​GTT​TTC​CTC​CTC​CGC​TC
Anpep	Forward: TGT​GTT​GGT​TAC​AGC​AGG​CA
Anpep	Reverse: TAT​CCT​CAC​AGG​GGG​TGG​AG
Gpnmb	Forward: CCG​GGC​ATA​CAT​TCC​CAT​CT
Gpnmb	Reverse: TGG​CAG​AGT​CGT​TGA​GGA​AG
Gmfg	Forward: AAA​TCC​GCA​CCA​CAG​ACG​AC
Gmfg	Reverse: GCC​AAA​GTT​GGA​ACT​GTA​GGG​A
Sel1l3	Forward: GGC​AGC​AAT​GGA​ATA​CGC​AG
Sel1l3	Reverse: AGA​CAC​AGG​CAG​GCA​TAA​GG
Slc39a4	Forward: GCA​GCA​GGG​GTT​TCC​AAA​AG
Slc39a4	Reverse: CCT​TGG​AAG​CAG​GAC​CCA​TT
Tnip3	Forward: CCG​CGA​GTC​AAC​AGA​GAC​AT
Tnip3	Reverse: ACT​CTG​TGT​GCT​CCA​TGC​AA
Slc13a3	Forward: TAC​CTG​GGG​GTG​GAG​CTA​TC
Slc13a3	Reverse: CTC​TGT​GCA​TGT​CCG​TCC​TT
Oas2	Forward: CCT​AAG​AGG​CTG​CTC​CGA​TG
Oas2	Reverse: TGC​CCT​GGT​ACA​GAC​ACT​TG
Marco	Forward: GAC​AAG​CCC​TTC​TTC​TCG​CT
Marco	Reverse: AGT​TGC​TCC​TGG​CTG​GTA​TG
Rhov	Forward: TCA​GCT​ACA​CCT​GCA​ATG​GG
Rhov	Reverse: GAG​AGA​ACG​AAG​CCG​GTC​AA
Lst1	Forward: AAG​GAA​TGC​CCA​GGT​CTC​AG
Lst1	Reverse: ACT​CAA​GTG​GGT​GTG​CTC​CT
Stra6l	Forward: GCC​CAC​AAG​AAG​GCG​AAA​AG
Stra6l	Reverse: AGG​TCC​CCG​TCT​CGT​TCA​TA

### 2.13 siRNA transfection

MC3T3-E1 and 3T3-L1 cells in good growth condition were plated at a specific density of 5 × 10^4 cells/mL in 12-well plates, with 1 mL per well. The plates were shaken gently to ensure uniform cell distribution. When the cells reached 60% confluence, the siRNA concentration was diluted with buffer, and 1×siRNA-mate plus transfection reagent (GenePharma, Shanghai) was added and mixed. The mixture was then added to the cells. After 72 h, the interference effect was detected by Western blot. The Cx3cr1 siRNA sequence was GAG​ACU​CUC​AAG​UUC​UAC​ATT UGU​AGA​ACU​UGA​GAG​UCU​CTT for 3T3-L1 and GGA​CAC​CUG​UAU​AGG​AAG​UTT ACU​UCC​UAU​ACA​GGU​GUC​CTT for MC3T3-E1, and the negative control was UUC​UCC​GAA​CGU​GUC​ACG​UTT ACG​UGA​CAC​GUU​CGG​AGA​ATT.

### 2.14 ALP, alizarin red and Oil Red O staining

BMMSCs and MC3T3-E1 cells were fixed with 4% paraformaldehyde at room temperature for 30 min, washed three times with PBS, and stained with an alkaline phosphatase staining kit working solution at room temperature for 30 min. After washing three times with PBS, images were captured using an inverted phase contrast microscope.

BMMSCs and MC3T3-E1 cells were fixed with 4% paraformaldehyde at room temperature for 30 min, washed twice with PBS, and stained with alizarin red staining solution at room temperature for 5 min. After washing three times with PBS, images were captured using an inverted phase contrast microscope.

BMMSCs and 3T3-L1 cells were fixed with 4% paraformaldehyde at room temperature for 30 min, washed twice with PBS, and stained with Oil Red O staining solution at room temperature for 30 min. After washing three times with PBS, images were captured using an inverted phase contrast microscope.

### 2.15 Statistical analysis

Data are presented as mean ± standard error of the mean (S.E.M.). All statistical analyses were performed using Prism 6.0 software (GraphPad Software, United States). A one-way ANOVA was used for statistical analysis of multiple comparisons, followed by Dunnett’s *post hoc* test to determine differences between the experimental groups and the designated control group. A p-value <0.05 was considered statistically significant between treatment groups.

## 3 Results

### 3.1 Effect of SC on ovariectomy-induced bone mass and bone metabolism

**FIGURE 1:**
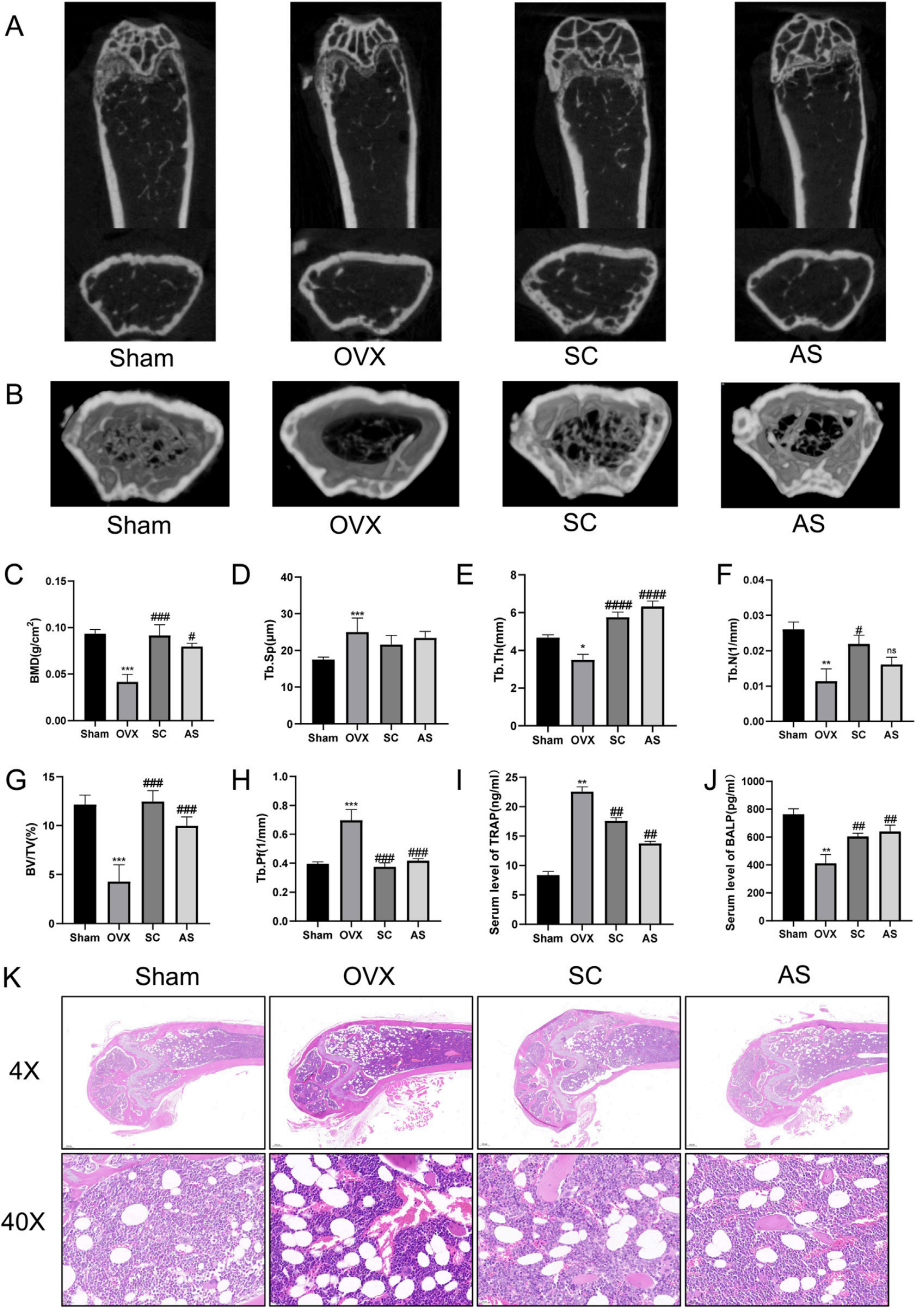
Effects of SC on Bone Microstructure in OVX Mice. **(A)** Micro-CT detection of femoral microstructure in each group of mice; **(B)** Three-dimensional reconstruction of bone microstructure in each group of mice; **(C–H)** BMD, Tb.Sp, Tb.Th, Tb.N, BV/TV, and Tb.Pf of mice in each group; **(I)** Determination of the expression level of serum TRAP by ELISA; **(J)** Determination of the expression level of serum BALP by ELISA. Compared with the Sham group, *P < 0.05, **P < 0.01, ***P < 0.001; compared with the OVX group, #P < 0.05, ^##^P < 0.01, ^###^P < 0.001, ^####^P < 0.0001; x ± s, n = 6; **(K)** H&E staining of femurs in each group of mice. Scale bar, 200 μm, 20 μm.

From the perspective of the characteristics of superclass distribution (Supplementary Figure S5), there are various categories of components with potential biological activities in the mixed extract of whole scorpions and centipedes. Phenylpropanoids and polyketides have a prominent proportion, accounting for 25.2% and 25.6%. Lipids and lipid-like molecules account for 22.7% and 19.8%. In addition, categories such as organoheterocyclic compounds and benzenoids also have distributions. These different superclass categories of components together form a complex chemical basis for the whole scorpion-centipede mixed system, providing diverse material sources for the exertion of its pharmacodynamic effects. To investigate the effect of the SC on bone mass and metabolism, we first examined the bone mineral density (BMD), bone volume/tissue volume (BV/TV), trabecular number (Tb.N), trabecular average thickness (Tb.Th), trabecular pattern factor (Tb.Pf), and trabecular separation (Tb.Sp) in OVX mice using micro-CT analysis. As shown in Figures 1A–H, the levels of BMD, BV/TV, Tb.Th, Tb.Sp, and Tb.N in the femur were significantly decreased, whereas Tb.Pf was significantly increased in the OVX mice compared with the sham controls. Importantly, treatment with the SC significantly reversed the OVX-induced decline in bone density and deterioration of bone microstructure when compared with the OVX group (Figures 1A–H). Besides, the markers related to osteoblastogenesis and osteoclastogenesis, BALP and TRAP, were selected to investigate the potential anti-osteoporotic effects of SC. As shown in Figures 1I,J, the serum level of BALP was significantly decreased, while the serum level of TRAP was significantly increased in the OVX mice compared with the sham group. Furthermore, the level of BALP was significantly increased, while the concentration of TRAP was significantly decreased in OVX mice treated with SC compared with the OVX controls (Figures 1I,J). Moreover, we conducted histomorphological analysis on OVX mice. Compared to the sham group, the cancellous bone in OVX mice was irregularly arranged, with fewer connections between them as well as a notable increase in adipose cells (Figure 1K). However, treatment with the SC resulted in a significant increase in trabecular bone volume and trabecular bone compactness, effectively mitigating the loss of trabecular networks, as well as a reduction in adipose cells when compared to the OVX controls (Figure 1K). These results indicate that SC could improve ovariectomy-induced bone mass and bone metabolism.

### 3.2 Effects of SC on osteogenic and adipogenic differentiation

**FIGURE 2:**
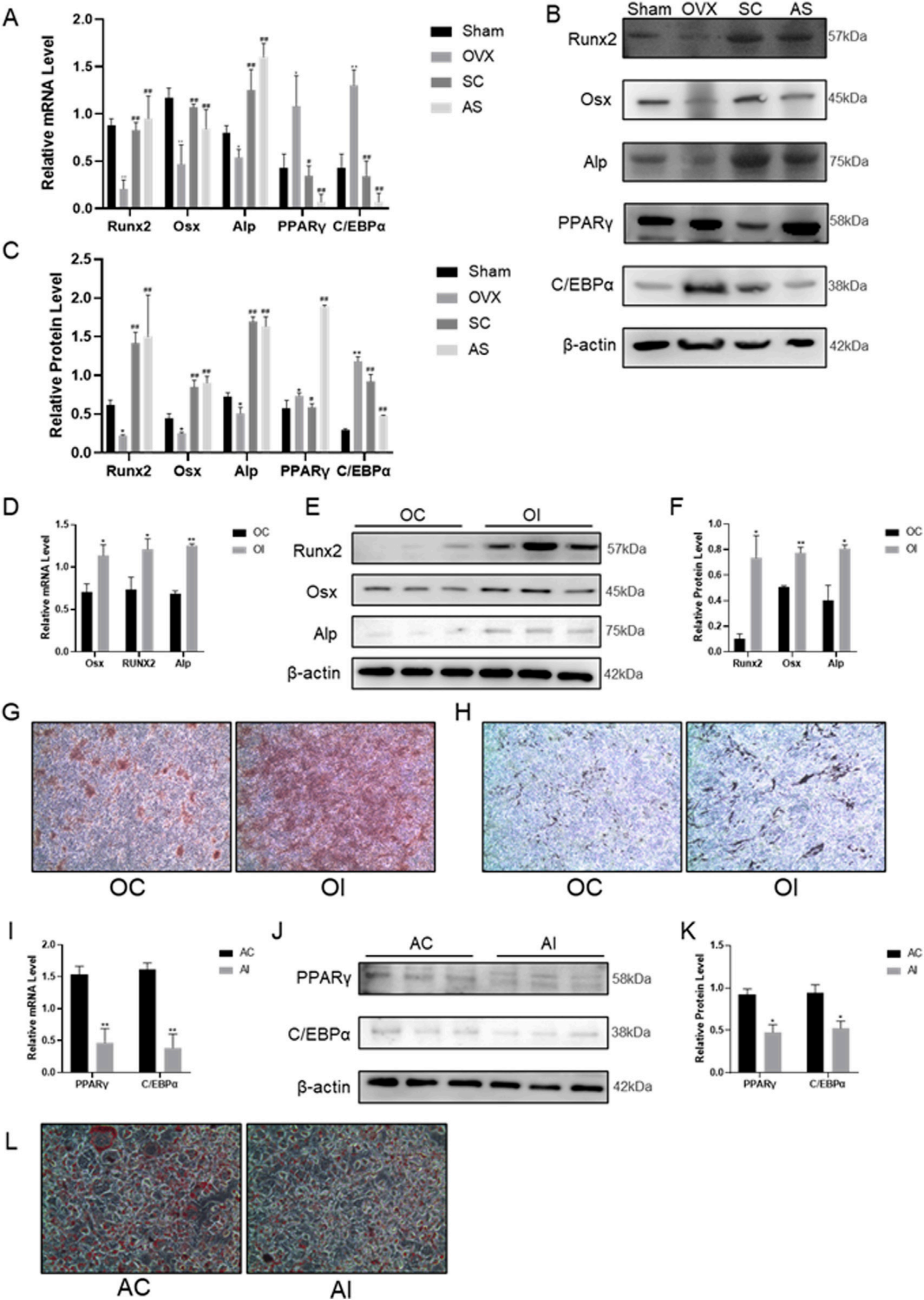
Effects of SC on Osteogenic and Adipogenic Differentiation. **(A–C)** qPCR, WB, and relative grayscale analysis of Runx2, Osx, Alp, PPARγ, and C/EBPα in mouse bone tissue; **(D–F)** qPCR, WB, and relative grayscale analysis of osteogenic differentiation markers Runx2, Osx, and Alp in BMMSCs after SC intervention; **(G)** Alizarin red staining of BMMSCs; **(H)** ALP activity test results of BMMSCs, scale bar, 50 μm. **(I–K)** qPCR, WB, and relative grayscale analysis of adipogenic differentiation markers PPARγ and C/EBPα in BMMSCs after SC intervention; **(L)** Oil red O staining of BMMSCs. Results are expressed as mean ± SD. n = 3, *P < 0.05, **P < 0.01, #P < 0.05, ##P < 0.01.

In order to evaluate the function of the SC in osteogenic and adipogenic differentiation, osteogenic and adipogenic genes were identified in bone tissue and BMMSCs. As shown in Figures 2A–C, the levels of osteogenic differentiation-related indicators such as ALP, Runx2, and Osx in the femur were significantly decreased, whereas adipogenic-related indicators PPARγ and C/EBPα were significantly increased in the OVX mice compared with the sham controls. Notably, SC treatment significantly increased the levels of the osteogenic indicators ALP, Runx2 and Osx in bone tissue, while simultaneously decreasing the levels of the adipogenic indicators PPARγ and C/EBPα, as compared to the OVX group.

*In vitro*, BMMSCs were extracted and induced to undergo osteogenic and adipogenic differentiation for 7 days following treatment with the SC for 24 h. Alizarin Red staining results showed that mineralized nodules were significantly increased in BMMSCs after treatment with the SC (Figures 2G,H). Additionally, ALP, Runx2 and Osx expression levels were significantly higher in BMMSCs treated with SC than in the control group (Figures 2D–F). In contrast, Oil Red O staining results showed that lipid droplet formation was significantly reduced in BMMSCs treated with the SC compared to the control group (Figure 2L). Moreover, the levels of PPARγ and C/EBPα expression were significantly decreased in BMMSCs treated with the SC compared to the control group (Figures 2I–K). Taken together, these results suggest that the SC promotes osteogenic differentiation and inhibits adipogenic differentiation.

### 3.3 RNA-seq sequencing to identify Cx3cr1-mediated the effect of SC on osteogenic and adipogenic differentiation

**FIGURE 3:**
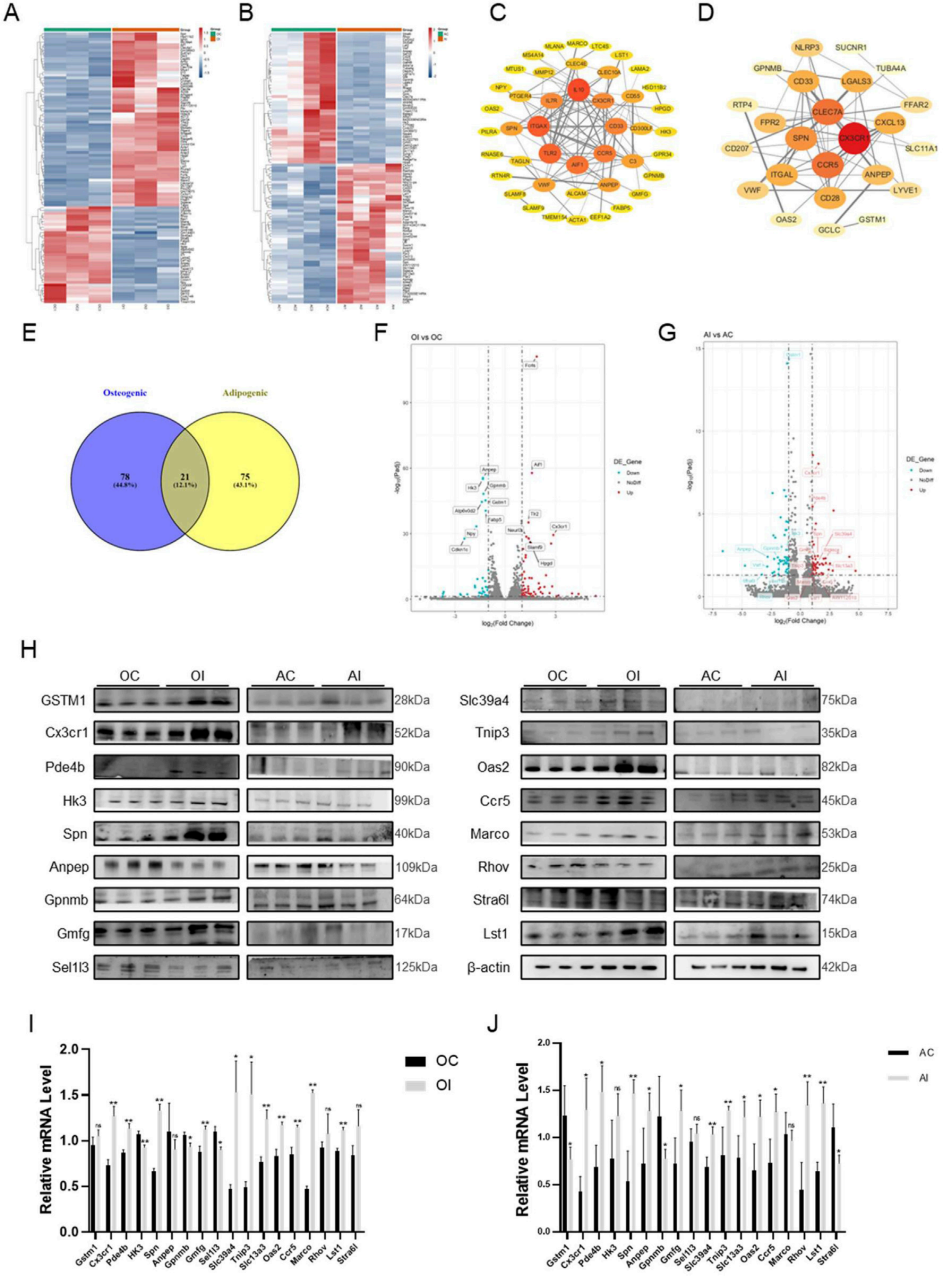
RNA-seq and Screening of BMMSCs after SC Intervention. **(A)** Heatmap of differential genes for osteogenic differentiation after SC intervention; **(B)** Heatmap of differential genes for adipogenic differentiation after SC intervention; **(C)** PPI protein interaction map of osteogenic differential genes; **(D)** PPI protein interaction map of adipogenic differential genes; **(E)** Venn diagram of cross-genes for osteogenic and adipogenic differentiation; **(F)** Volcano plot of common differential genes for osteogenic differentiation; **(G)** Volcano plot of common differential genes for adipogenic differentiation; **(H)** WB analysis and screening of differential genes; **(I,J)** qPCR analysis and screening of differential genes. n = 3, *P < 0.05, **P < 0.01.

To explore the mechanism of centipede-scorpion in regulating osteogenic and adipogenic differentiation, we conducted a transcriptome analysis of BMMSCs treated with the SC. Firstly, BMSCs were induced to undergo osteogenic and adipogenic differentiation for 7 days, respectively. Then, transcriptome analysis was performed on these cells after 24 h of SC treatment. The results showed 99 differentially expressed genes (DEGs) in osteogenic differentiation and 96 DEGs in adipogenic differentiation (Figures 3A,B). We further constructed and analyzed protein-protein interaction (PPI) subnetworks for the osteogenic and adipogenic DEGs, respectively, using STRING to build the network and visualizing it in Cytoscape 3.10.1. By ranking the genes based on their degree, we identified key osteogenic targets, including IL10, ITGAX, TLR2, AIF1, CCR5, CD33, IL7R, and Cx3cr1, as well as key adipogenic targets such as Cx3cr1, CLEC7A, CCR5, and SPN (Figures 3C,D). We then performed a cross-comparison between the two sets of targets and identified 21 overlapping DEGs (Figures 3E–G). Subsequently, the protein and mRNA assays were conducted to exclude false-positive results (Figures 3H–J). Ultimately, Cx3cr1 was determined to be the key regulator of the osteogenic and adipogenic differentiation of BMMSCs.

### 3.4 SC increased osteogenic differentiation and inhibited adipogenic differentiation through promoting Cx3cr1 expression

**FIGURE 4:**
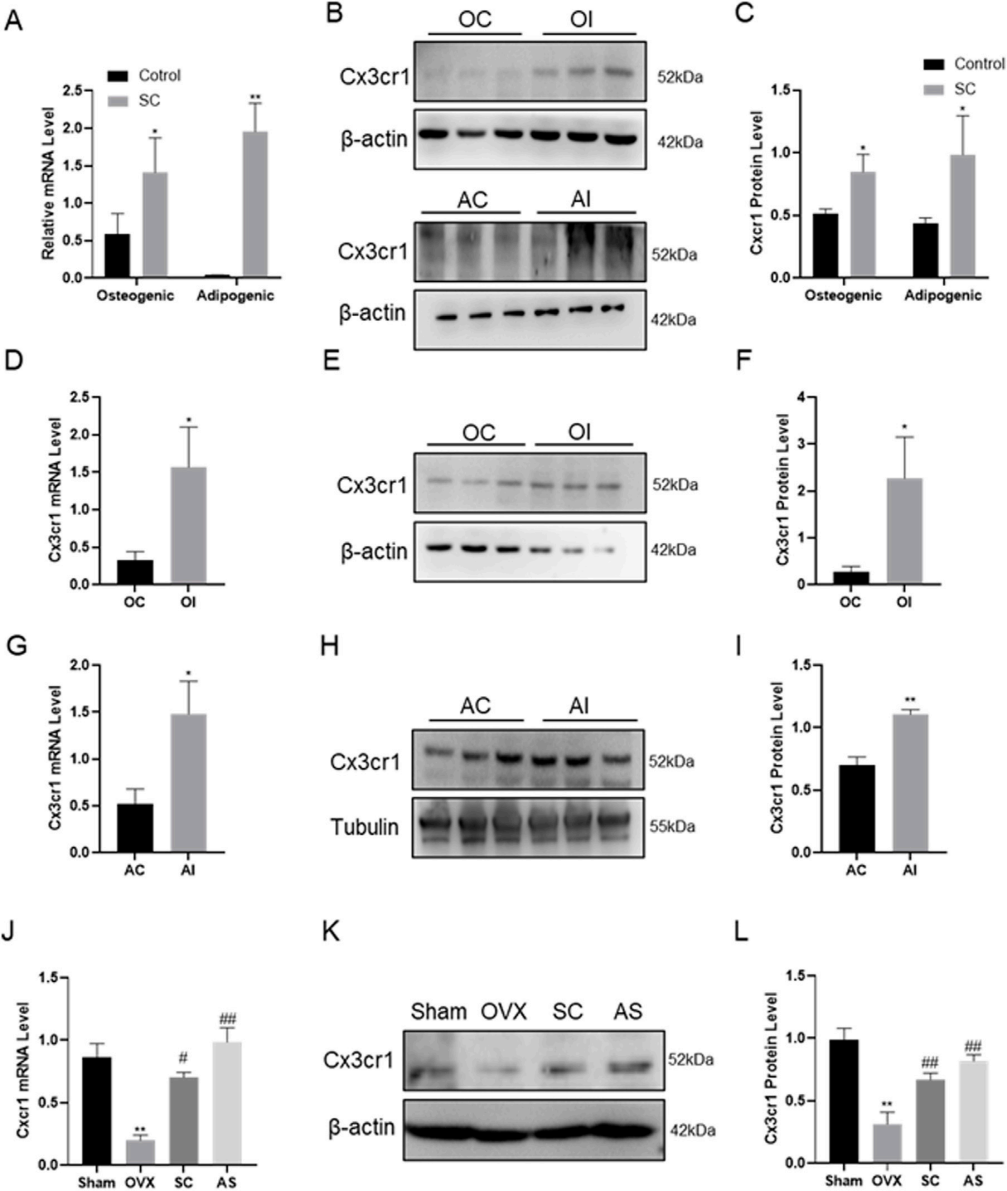
Gene Expression of Cx3cr1 after SC Intervention. **(A–C)** qPCR, WB, and relative grayscale analysis of Cx3cr1 in BMMSCs after SC intervention; **(D–F)** qPCR, WB, and relative grayscale analysis of Cx3cr1 in MC3T3-E1 after SC intervention; **(G–I)** qPCR, WB, and relative grayscale analysis of Cx3cr1 in 3T3-L1 after SC intervention; **(J–L)** qPCR, WB, and relative grayscale analysis of Cx3cr1 in bone tissue after SC intervention. n = 3, *P < 0.05, **P < 0.01, #P < 0.05, ##P < 0.01.

**FIGURE 5:**
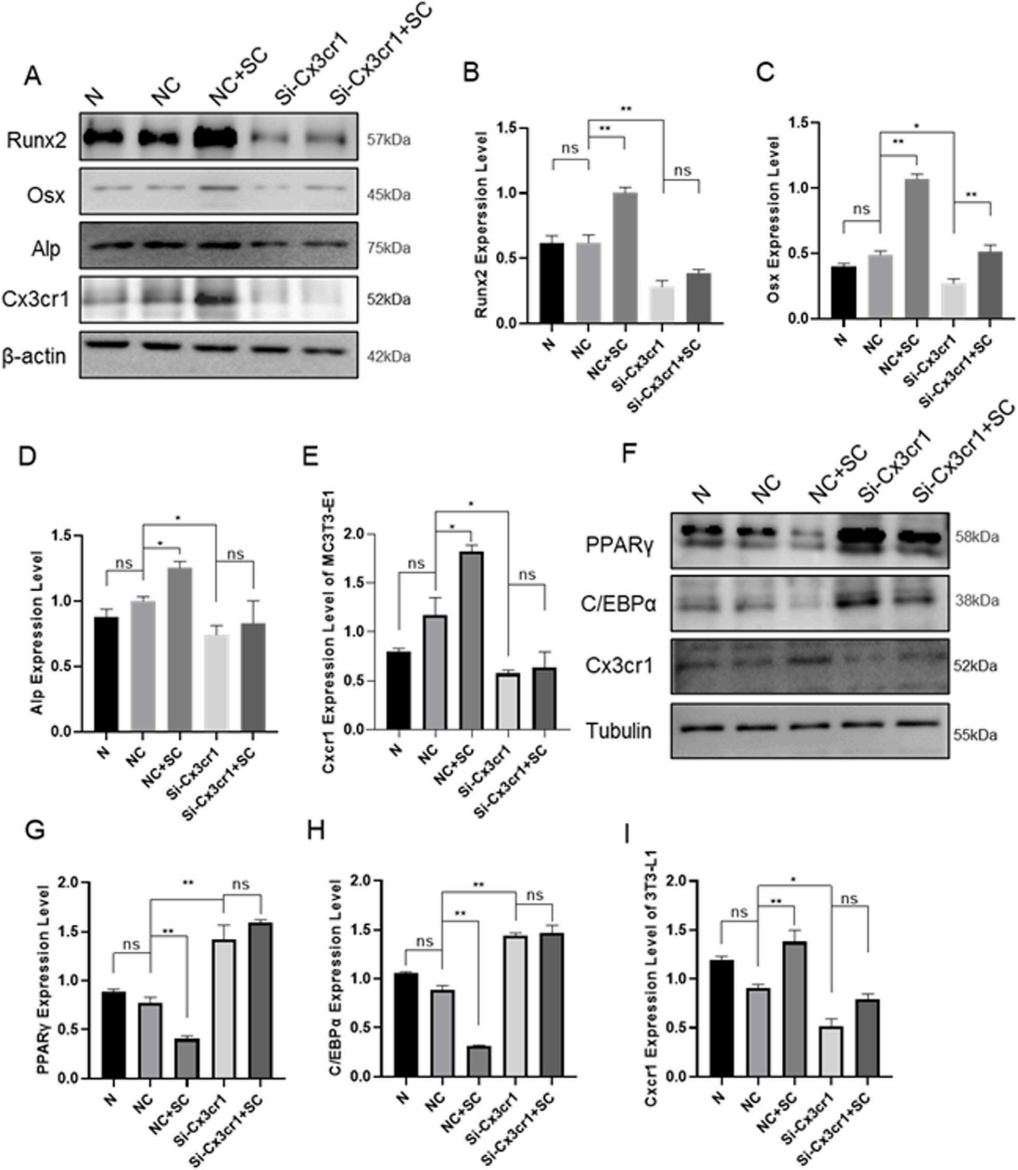
Cx3cr1 Knockdown Inhibits the Effects of SC on Osteogenic and Adipogenic Differentiation of MC3T3-E1 and 3T3-L1. **(A–E)** WB and relative grayscale analysis of osteogenic differentiation proteins Runx2, Osx, and Alp in MC3T3-E1 after Cx3cr1 knockdown; **(F–I)** WB and relative grayscale analysis of adipogenic differentiation proteins PPARγ and C/EBPα in 3T3-L1 after Cx3cr1 knockdown. n = 3, *P < 0.05, **P < 0.01.

To validate the effect of SC on osteogenic and adipogenic differentiation via Cx3cr1, the Cx3cr1 and osteogenic and adipogenic genes were detected in bone tissue, BMMSCs, MC3T3-E1, and 3T3-L1 cells. As shown in Figures 4A–L, SC treatment significantly increased Cx3cr1 expression in BMMSCs, MC3T3-E1, and 3T3-L1 cells as well as bone tissue of OVX mice. We further investigated osteogenic and adipogenic genes in Cx3cr1 knockdown MC3T3-E1 and 3T3-L1 cells. As shown in Figures 5A–E, the SC-induced elevation of Runx2, Osx, and Alp expression in MC3T3-E1 cells was blocked when Cx3cr1 was knocked down in MC3T3-E1 cells. Besides, the SC-induced reduction of PPARγ and C/EBPα expression in 3T3-L1 cells was blocked when Cx3cr1 was knocked down in 3T3-L1 cells (Figures 5F–I). These results suggest that Cx3cr1 mediates the effects of SC on osteogenic and adipogenic differentiation.

## 4 Discussion

In bone rejuvenation, new bone is formed (bone formation) and old bone is removed (bone resorption) (Eastell and Szulc, 2017). Osteoclasts remove old bone from various skeletal locations during bone resorption. Osteoblasts then appear at the resorption sites to begin the process of bone formation. However, the equilibrium between bone resorption and bone creation is upset after menopause, with bone resorption outpacing bone formation as a result of hormonal imbalances, age, oxidative stress, and other reasons. This causes PMOP to develop gradually. Current PMOP treatments aim to restore normal bone remodelling, boost bone density and reduce the risk of fractures (Ansari et al., 2023). The activity of bone-resorbing cells (osteoclasts) can be reduced using bisphosphonates such as alendronate (Ansari et al., 2023). Hormone replacement therapy (HRT) and other oestrogen-like agents, such as the selective oestrogen receptor modulator (SERM) raloxifene, are effective in preventing bone loss (Ansari et al., 2023). Traditional Chinese medicine primarily uses herbal remedies to nourish the liver and kidneys in the treatment of PMOP (Zeng, 2023). The scorpion-centipede (SC) is a traditional Chinese herbal medicine pair that is used to treat PMOP, but its precise mechanism of action has not yet been determined. In this study, we found that the SC effectively increases bone mass and osteogenesis by promoting Cx3cr1, thereby increasing osteogenic differentiation and inhibiting adipogenic differentiation in BMMSCs.

SC, a traditional Chinese medicine, has been widely used in traditional Chinese medicine, particularly for treating rheumatoid arthritis (Liu et al., 2012), asthma (Zhang et al., 2023), epilepsy (Rong et al., 2021), and various intractable pains. In recent years, with the deepening research on postmenopausal osteoporosis (PMOP), an increasing number of clinical cases and observational studies have shown that SC exhibits definite clinical efficacy in the treatment of PMOP. They can effectively alleviate patients’ bone pain symptoms, increase bone mineral density (BMD), reduce the risk of fractures, and thereby improve patients’ quality of life (Min et al., 2010). Studies have reported that venom extracts from certain scorpions can restore serum levels of serum ALP, TRAP, PTH, osteocalcin, TNF-α, and bone minerals in OVX rats, combatting osteoporosis and enhancing BMD (Gomes et al., 2009; Haldar et al., 2010; Uzair et al., 2018). Consistent with these findings, we found that SC increased serum BALP levels and femoral BMD, BV/TV, Tb.Th and Tb.N in OVX mice, while decreasing Tb.Pf and serum TRAP levels. This indicates that SC promotes bone mass and metabolism. Meanwhile, we found that SC decreased bone marrow cavity and fat vacuole development, while increasing trabecular bone volume and compactness. This finding supports the idea that SC promotes bone formation. Taken together, these findings demonstrate that SC significantly increases bone mass production and prevents osteoporosis brought on by a decrease in estrogen.

BMMSCs are the primary source of bone formation. BMMSCs are pluripotent stem cells that can develop into adipocytes, chondroblasts, osteoblasts, and other cell types. According to clinical observations, the growth of bone marrow adipose tissue (BMAT) frequently occurs alongside PMOP. Hormone insufficiency caused by ovariectomy has also been shown in animal tests to increase BMAT volume and reduce cortical and trabecular bone mass. Additionally, elevated BMAT levels have been found to stimulate osteoclast-mediated bone resorption and suppress osteoblast-mediated bone formation via paracrine pathways, thereby disrupting bone homeostasis (Li et al., 2024). Thus, one of the key reasons for PMOP is the increased differentiation of BMMSCs into adipocytes and the reduced differentiation of BMMSCs into osteoblasts. Runx2, Osx, and Alp are among the three important factors influencing osteoblast development from BMMSCs, while PPARγ and C/EBPα are key adipogenic-related genes influencing adipose development from BMMSCs (Kokabu et al., 2016). Previous studies have proposed that SC promotes osteoblast and BMMSC development and suppresses osteoclast differentiation through elevating the β-catenin expression (Yuan et al., 2018). Consistent with this, our study revealed that SC treatment resulted in higher Runx2, Osx, and Alp mRNA and protein expression in the bone tissues and BMMSCs of OVX mice. Furthermore, we found that SC treatment decreased the expression of PPARγ and C/EBPα in the bone tissues and BMMSCs of OVX mice. Collectively, these findings indicate that SC promotes osteogenic differentiation and inhibits adipogenic differentiation, thereby increasing bone formation in POMP.

Previous studies have confirmed that SC can regulate the osteogenic and adipogenic differentiation of BMMSCs. We performed transcriptome sequencing on BMMSCs undergoing osteogenic and adipogenic differentiation with SC treatment. The screening results showed that a highly correlated potential regulatory target, CX3CR1 (a chemokine receptor), was identified. C-X3-C motif chemokine receptor 1 (Cx3cr1) belongs to the G-protein-coupled receptor superfamily and is the only receptor that is specific to CX3C chemokine ligand 1 (CX3CL1) (Zhuang et al., 2019; Subbarayan et al., 2022). Numerous studies have confirmed that the Cx3cr1 activity is closely associated with the pathogenesis of osteoporosis (Wojdasiewicz et al., 2019). Studies have shown that, during the osteogenic differentiation of BMMSCs, the ligand Cx3cl1 for Cx3cr1 continuously increases. This suggests that CX3CL1 may promote the formation of an osteogenic microenvironment by regulating cell distribution and aggregation, facilitating cell-cell induction, and paracrine signaling (Xiao et al., 2009). Simultaneously, impaired osteoblast differentiation and function have been observed in Cx3cr1-deficient mice, suggesting that Cx3cr1 deficiency may disrupt the chemokine network by altering the spatiotemporal input of chemokine signals to osteoblasts. This could result in the disordered temporal expression of osteogenic transcription factors and bone matrix proteins, subsequently impairing mineral deposition. Furthermore, stimulating osteoblasts with CX3CL1 increases the levels of differentiation-related markers Runx2 and Osx, demonstrating the crucial role of the Cx3cr1-Cx3cl1 axis in regulating osteoblast differentiation (Hoshino et al., 2013). Additionally, Cx3cr1 plays a definite role in adipogenic differentiation. Several studies have shown that high levels of PPARγ, which plays a vital role in adipogenic differentiation, can reduce the expression of Cx3cr1 and its upstream chemokine Cx3cl1 (Wan and Evans, 2010; Zhang et al., 2012). In the current study, we found that SC treatment increased the mRNA and protein levels of Cx3cr1 in bone tissue, BMMSCs, MC3T3-E1, and 3T3-L1 cells. Specifically, we found that SC-induced increases in Runx2, Osx and Alp expression in MC3T3-E1 cells were blocked when Cx3cr1 was knocked down in these cells. Furthermore, the SC-induced reduction of PPARγ and C/EBPα expression in 3T3-L1 cells was abolished when Cx3cr1 was knocked down in these cells. These results suggest that Cx3cr1 mediates the effects of SC on osteogenic and adipogenic differentiation, thereby improving PMOP. While this study reveals the effects of SC on osteogenic and adipogenic differentiation, the experimental design has some limitations. For example, we only observed changes in gene expression and related indicators after SC treatment, rather than exploring how SC regulates Cx3cr1 expression or the specific mechanisms through which these changes affect osteogenic and adipogenic differentiation. Besides, after silencing Cx3cr1, osteogenic capacity was not assessed through functional assays such as Alizarin Red staining and ALP activity measurement. These require further experimental validation.

Moreover, the pathogenesis of PMOP is characterized by high complexity and multidimensionality, and its specific mechanisms require further in-depth exploration. For example, in the regulation of osteogenic differentiation, the stability of the key osteogenic receptor FGFR2 depends on the inhibition of the E3 ubiquitin ligase SMURF1 by the deubiquitinating enzyme OTUB1. Deficiency of OTUB1 can lead to excessive degradation of FGFR2 through the lysosomal pathway, thereby inhibiting the osteogenic differentiation process (Zhu et al., 2023). Additionally, extracellular vesicles (EVs), as important mediators of intercellular communication, can directly influence the differentiation fate of BMMSCs when their cargo components (such as pro-osteogenic miR-935 and anti-osteogenic miR-214-3p) are abnormally altered (Fang et al., 2024). Meanwhile, differences in the surface targeting properties of EVs can significantly affect their regulatory efficiency on bone tissue. On the other hand, small extracellular vesicles (sEVs) derived from synovial fibroblasts can deliver miRNA15-29148 to downregulate the expression of the anti-apoptotic gene CIAPIN1, thereby exacerbating chondrocyte apoptosis and disrupting the bone microenvironment. This mechanism may also be involved in the inhibition of osteogenic function in BMMSCs (Zhang et al., 2025). Recent studies also suggest that activation of the glycolysis–lactate–histone lactylation axis in endothelial cells (ECs) can enhance the level of histone H3K18 lactylation (H3K18la) in BMMSCs through lactate secretion, directly regulating the transcription of osteogenic genes such as COL1A2 and COMP (Wu et al., 2023). The imbalance in this metabolic–epigenetic crosstalk is also an important contributing factor to bone loss in PMOP. Therefore, there remains broad scope for further research into the mechanisms of PMOP. In-depth exploration of the above directions will not only help systematically elucidate the multi-target and multi-pathway regulatory network of SC in intervening in PMOP but will also enhance the understanding of the complex pathogenesis of PMOP, thereby providing new theoretical foundations and insights for the development of targeted and comprehensive clinical treatment strategies.

Although the single-dose design adopted in this study cannot fully elaborate the dose-effect relationship and long-term action characteristics of SC, with certain limitations, it is a reasonable choice based on the core positioning of the study and experimental ethical requirements. Firstly, this study focuses on the effect of SC in OVX mouse models. Currently, there is a lack of basic activity data of SC in this model in the field, so this study is typical early-stage exploratory research. Its core objective is to verify whether SC has a clear biological response and acute safety, rather than directly quantifying the effect differences under different doses. In the absence of previous reference data, the single-dose design can confirm the feasibility of this research direction with the lowest research cost, effectively avoiding the waste of experimental resources that may be caused by the blindness of dose setting in multi-dose designs. Additionally, current research on SC for the treatment of PMOP is almost blank in the field. The single-dose experimental results obtained in this study can not only fill this research gap but also provide a critical basic dose range and safety boundary for subsequent multi-dose studies, thus possessing irreplaceable early exploratory value. In the future, based on the single-dose results of this study, we will further design follow-up experiments including three dose gradients to explore the dose-effect relationship in depth and make up for the limitations of the current design.

## 5 Conclusion and future perspectives

In summary, our research showed that SC protects osteoporosis by dramatically increasing bone mass and bone metabolism. Furthermore, this study demonstrates that SC effectively promotes Cx3cr1, thereby improving osteogenic differentiation and inhibiting adipogenic differentiation. This boosts bone mass and osteogenesis. These findings enhance the mechanisms of SC and its potential to treat PMOP. These findings amplified the mechanisms of the SC and its potential to treat PMOP. Although this study highlights the potential mechanism by which Cx3cr1 mediates SC regulation of PMOP, none of the animal models used in this study were Cx3cr1 gene knockout. In future, we will validate SC regulation of PMOP through Cx3cr1 using Cx3cr1 gene knockout animal models.

## Data Availability

The original contributions presented in the study are publicly available. This data can be found here: https://ngdc.cncb.ac.cn/gsa/browse/CRA031108 (GSA: CRA031108).
