# Supplementary material for: Scorpion-Centipede extracts mitigate ovariectomy-induced osteoporosis in mice through facilitating Cx3cr1 expression
Source: Front Pharmacol. 2025 Oct 24;16:1604096. doi: 10.3389/fphar.2025.1604096 (PMC12592035; doi:10.3389/fphar.2025.1604096)
Supplement: Supplementary file 1 [file Supplementaryfile1.docx]

Supplementary Data

| Table S1. Composition of SC | | | | |
| --- | --- | --- | --- | --- |
| Pharmaceutical name | Botanical or zoological name | Family and plant part use | Chinese name | % (w/w) |
| Scorpion | Buthus martensii Karsch | Buthidae; dry polypite | Quan Xie | 50 |
| Centipede | Scolopendra subspinipes mutilans L. Koch | Scolopendra; dry polypite | Wu Gong | 50 |
| The zoological names are from The Pharmacopoeia of the People's Republic of China (2020 Edition). | | | | |

**Table S2. Vanquish system used for LC-MS analysis of SC**

| Time(min) | Flow-rate(μl /min) | A% | B% |
| --- | --- | --- | --- |
| 0.00 | 400 | 95 | 5 |
| 1.50 | 400 | 95 | 5 |
| 2.50 | 400 | 90 | 10 |
| 14.00 | 400 | 60 | 40 |
| 24.00 | 400 | 5 | 95 |
| 27.00 | 400 | 5 | 95 |
| 27.10 | 400 | 95 | 5 |
| 30.00 | 400 | 95 | 5 |

**Table S3. Ingredient separation and identiffcation of SC by LC-MS.**

| NO. | tR (min) | Name | Formula | Ion species | Precursor m/z |
| --- | --- | --- | --- | --- | --- |
| 1 | 0.50497 | Spermidine | C7H19N3 | [M+H]+ | 146.164 |
| 2 | 16.9231 | Piperine | C17H19NO3 | [M+H]+ | 286.142 |
| 3 | 0.84833 | Xanthine | C5H4N4O2 | [M+H]+ | 153.039 |
| 4 | 2.07355 | Phenethylamine | C8H11N | [M+H]+ | 122.096 |
| 5 | 2.51875 | Lycopsamine | C15H25NO5 | [M+H]+ | 300.181 |
| 6 | 5.34455 | Riboflavin | C17H20N4O6 | [M+H]+ | 377.141 |
| 7 | 0.59898 | L-Arginine | C6H14N4O2 | [M+H]+ | 175.118 |
| 8 | 21.2665 | Vaccenic acid | C18H34O2 | [M+H]+ | 283.266 |
| 9 | 7.59317 | 2,2-Dimethyl-2,3-dihydro-1-benzofuran-7-amine | C10H13NO | [M+H]+ | 164.109 |
| 10 | 3.44195 | Agmatine | C5H14N4 | [M+H]+ | 131.131 |
| 11 | 17.5905 | Imperatorin | C16H14O4 | [M+H]+ | 271.099 |
| 12 | 0.81715 | Uracil | C4H4N2O2 | [M+H]+ | 113.035 |
| 13 | 0.78582 | Guanine | C5H5N5O | [M+H]+ | 152.056 |
| 14 | 20.4295 | Cafestol | C20H28O3 | [M+H]+ | 317.215 |
| 15 | 12.0394 | Genistein | C15H10O5 | [M+H]+ | 271.061 |
| 16 | 0.84833 | Methionine | C5H11NO2S | [M+H]+ | 150.058 |
| 17 | 0.9105 | Inosine | C10H12N4O5 | [M+H]+ | 269.086 |
| 18 | 3.47312 | 4-hydroxybenzoic acid | C7H6O3 | [M+H]+ | 139.041 |
| 19 | 11.7282 | Icariin | C33H40O15 | [M+H]+ | 677.237 |
| 20 | 19.5864 | Carpachromene | C20H16O5 | [M+H]+ | 337.108 |
| 21 | 17.5905 | Aurantiamide acetate | C27H28N2O4 | [M+H]+ | 445.208 |
| 22 | 1.28785 | Pilocarpine | C11H16N2O2 | [M+H]+ | 209.129 |
| 23 | 25.1344 | VITAMIN D3 | C27H44O | [M+H]+ | 385.35 |
| 24 | 0.9105 | Guanosine | C10H13N5O5 | [M+H]+ | 284.103 |
| 25 | 2.39323 | KOJIC ACID | C6H6O4 | [M+H]+ | 143.035 |
| 26 | 20.6768 | Dibutyl phthalate | C16H22O4 | [M+H]+ | 279.159 |
| 27 | 7.31548 | 13a-Hydroxylupanin | C15H24N2O2 | [M+H]+ | 265.193 |
| 28 | 19.7756 | LINOLENIC ACID | C18H30O2 | [M+H]+ | 279.234 |
| 29 | 23.76 | Linolenic Acid ethyl ester | C20H34O2 | [M+H]+ | 307.262 |
| 30 | 4.0158 | Harmaline | C13H14N2O | [M+H]+ | 215.121 |
| 31 | 12.5283 | Quinine | C20H24N2O2 | [M+H]+ | 325.195 |
| 32 | 20.8928 | Cortisone | C21H28O5 | [M+H]+ | 361.201 |
| 33 | 22.2297 | Abietic acid | C20H30O2 | [M+H]+ | 303.23 |
| 34 | 0.50497 | Pyrrolidine | C4H9N | [M+H]+ | 72.0804 |
| 35 | 12.4208 | Peimisine | C27H41NO3 | [M+H]+ | 428.318 |
| 36 | 12.9758 | Angelol A | C20H24O7 | [M+H]+ | 377.162 |
| 37 | 7.2465 | Harpagide | C15H24O10 | [M+H]+ | 365.148 |
| 38 | 20.986 | Dihydropalmatine | C21H23NO4 | [M+H]+ | 354.167 |
| 39 | 23.2863 | Chaulmoogric Acid | C18H32O2 | [M+H]+ | 281.247 |
| 40 | 4.43183 | Vanillic acid | C8H8O4 | [M+H]+ | 169.048 |
| 41 | 4.75768 | Heliotrine | C16H27NO5 | [M+H]+ | 314.197 |
| 42 | 4.20712 | N-acetyltyramine | C10H13NO2 | [M+H]+ | 180.104 |
| 43 | 16.0938 | 2''-O-rhamnosyl icariside II | C33H40O14 | [M+H]+ | 661.251 |
| 44 | 19.1129 | Dehydroabietic acid | C20H28O2 | [M+H]+ | 301.218 |
| 45 | 13.1123 | Cyclopamine | C27H41NO2 | [M+H]+ | 412.317 |
| 46 | 11.1613 | Dihydrocapsaicin | C18H29NO3 | [M+H]+ | 308.223 |
| 47 | 23.069 | Arachidonic acid | C20H32O2 | [M+H]+ | 305.248 |
| 48 | 0.9105 | Adenosine | C10H13N5O4 | [M+H]+ | 268.106 |
| 49 | 20.986 | Steviol | C20H30O3 | [M+H]+ | 319.229 |
| 50 | 0.53648 | Spermine | C10H26N4 | [M+H]+ | 203.221 |
| 51 | 1.22417 | Honokiol | C18H18O2 | [M+H]+ | 267.136 |
| 52 | 10.2901 | Berberine | C20H17NO4 | [M+H]+ | 336.125 |
| 53 | 20.7078 | Phthalic anhydride | C8H4O3 | [M+H]+ | 149.023 |
| 54 | 23.4418 | brassinolide | C28H48O6 | [M+H]+ | 481.352 |
| 55 | 0.63015 | Tanshinone I | C18H12O3 | [M+H]+ | 277.088 |
| 56 | 0.66148 | Bilobalide | C15H18O8 | [M+H]+ | 327.112 |
| 57 | 8.39238 | Deoxyvasicinone | C11H10N2O | [M+H]+ | 187.087 |
| 58 | 5.47605 | Tetrahydrocolumbamine | C20H23NO4 | [M+H]+ | 342.173 |
| 59 | 1.16117 | Isorhynchophylline | C22H28N2O4 | [M+H]+ | 385.217 |
| 60 | 2.55025 | isoferulic acid | C10H10O4 | [M+H]+ | 195.067 |
| 61 | 21.4532 | DIOSGENIN | C27H42O3 | [M+H]+ | 415.325 |
| 62 | 2.59207 | Mitragynine | C23H30N2O4 | [M+H]+ | 399.233 |
| 63 | 8.1502 | VINPOCETINE | C22H26N2O2 | [M+H]+ | 351.21 |
| 64 | 13.4881 | Deoxycorticosterone | C21H30O3 | [M+H]+ | 331.228 |
| 65 | 12.6313 | alpha-Santonin | C15H18O3 | [M+H]+ | 247.131 |
| 66 | 25.0397 | Korseveriline | C27H45NO3 | [M+H]+ | 432.346 |
| 67 | 20.3987 | Denudatine | C22H33NO2 | [M+H]+ | 344.254 |
| 68 | 14.1662 | BRUCINE | C23H26N2O4 | [M+H]+ | 395.195 |
| 69 | 4.30217 | Corydaline | C22H27NO4 | [M+H]+ | 370.202 |
| 70 | 24.5464 | Lappaconitine | C32H44N2O8 | [M+H]+ | 585.313 |
| 71 | 2.65937 | Cinchonine | C19H22N2O | [M+H]+ | 295.177 |
| 72 | 1.16117 | strychnine N-oxide | C21H22N2O3 | [M+H]+ | 351.168 |
| 73 | 8.82705 | leonurine | C14H21N3O5 | [M+H]+ | 312.159 |
| 74 | 22.3861 | Jervine | C27H39NO3 | [M+H]+ | 426.3 |
| 75 | 14.8619 | Viridiflorine | C15H27NO4 | [M+H]+ | 286.199 |
| 76 | 6.17773 | Suberic acid | C8H14O4 | [M-H]- | 173.08 |
| 77 | 8.20352 | Azelaic acid | C9H16O4 | [M-H]- | 187.096 |
| 78 | 10.2173 | Sebacic acid | C10H18O4 | [M-H]- | 201.113 |
| 79 | 0.88565 | Xanthine | C5H4N4O2 | [M-H]- | 151.028 |
| 80 | 23.1156 | Arachidonic acid | C20H32O2 | [M-H]- | 303.236 |
| 81 | 0.79212 | Pyroglutamic acid | C5H7NO3 | [M-H]- | 128.034 |
| 82 | 0.88565 | Citric acid | C6H8O7 | [M-H]- | 191.023 |
| 83 | 3.59688 | Gentisic acid | C7H6O4 | [M-H]- | 153.018 |
| 84 | 1.58165 | Glutaric acid | C5H8O4 | [M-H]- | 131.034 |
| 85 | 2.09053 | Protocatechuic acid | C7H6O4 | [M-H]- | 153.018 |
| 86 | 0.82363 | Uric acid | C5H4N4O3 | [M-H]- | 167.023 |
| 87 | 7.36862 | Salicylic acid | C7H6O3 | [M-H]- | 137.026 |
| 88 | 5.85605 | p-Coumaric acid | C9H8O3 | [M-H]- | 163.042 |
| 89 | 3.52808 | 4-hydroxybenzoic acid | C7H6O3 | [M-H]- | 137.026 |
| 90 | 0.57195 | L-Arginine | C6H14N4O2 | [M-H]- | 173.102 |
| 91 | 12.0134 | Apigenin | C15H10O5 | [M-H]- | 269.045 |
| 92 | 18.7142 | Carnosol | C20H26O4 | [M-H]- | 329.174 |
| 93 | 0.82363 | Lactic acid | C3H6O3 | [M-H]- | 89.0241 |
| 94 | 22.2744 | Glycocholic Acid | C26H43NO6 | [M-H]- | 464.307 |
| 95 | 5.31702 | Riboflavin | C17H20N4O6 | [M-H]- | 375.126 |
| 96 | 4.60668 | Benzoic acid | C7H6O2 | [M-H]- | 121.031 |
| 97 | 0.88565 | Inosine | C10H12N4O5 | [M-H]- | 267.077 |
| 98 | 3.59688 | Gallic acid | C7H6O5 | [M-H]- | 169.013 |
| 99 | 6.59075 | ferulic acid | C10H10O4 | [M-H]- | 193.048 |
| 100 | 22.3677 | LINOLENIC ACID | C18H30O2 | [M-H]- | 277.216 |
| 101 | 0.60412 | FRUCTOSE | C6H12O6 | [M-H]- | 179.056 |
| 102 | 3.95278 | 4-HYDROXYPHENYL ACETIC ACID | C8H8O3 | [M-H]- | 151.042 |
| 103 | 0.91713 | Ascorbic acid | C6H8O6 | [M-H]- | 175.023 |
| 104 | 0.57195 | L-Histidine | C6H9N3O2 | [M-H]- | 154.061 |
| 105 | 0.72913 | Uracil | C4H4N2O2 | [M-H]- | 111.021 |
| 106 | 23.924 | Embelin | C17H26O4 | [M-H]- | 293.179 |
| 107 | 20.4531 | Ricinoleic acid | C18H34O3 | [M-H]- | 297.241 |
| 108 | 19.1361 | ISOSTEVIOL | C20H30O3 | [M-H]- | 317.212 |
| 109 | 0.69778 | Hypoxanthine | C5H4N4O | [M-H]- | 135.031 |
| 110 | 0.60412 | 5-Methoxypsoralen | C12H8O4 | [M-H]- | 215.033 |
| 111 | 5.91955 | trans-Cinnamate | C9H8O2 | [M-H]- | 147.045 |
| 112 | 22.2744 | Neoabietic acid | C20H30O2 | [M-H]- | 301.215 |
| 113 | 1.26648 | Thymidine | C10H14N2O5 | [M-H]- | 241.086 |
| 114 | 13.0829 | Madecassoside | C48H78O20 | [M-H]- | 973.505 |
| 115 | 23.0846 | Osajin | C25H24O5 | [M-H]- | 403.154 |
| 116 | 23.1156 | Corosolic acid | C30H48O4 | [M-H]- | 471.35 |
