# Supplementary material for: Scorpion-Centipede extracts mitigate ovariectomy-induced osteoporosis in mice through facilitating Cx3cr1 expression
Source: Front Pharmacol. 2025 Oct 24;16:1604096. doi: 10.3389/fphar.2025.1604096 (PMC12592035; doi:10.3389/fphar.2025.1604096)
Supplement: Supplementary file 2 [file Supplementaryfile2.docx]

Supplementary Data


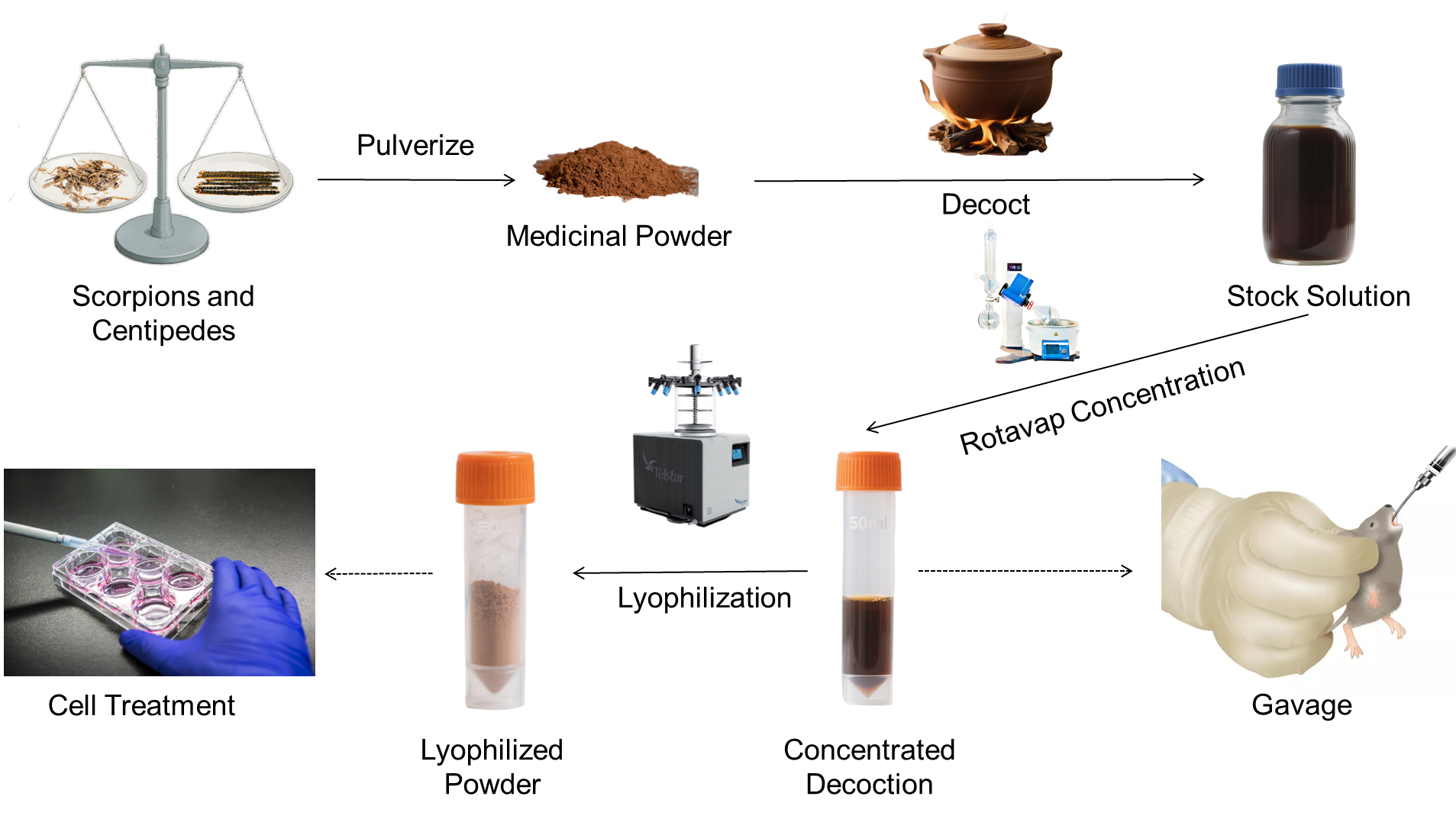


Figure S1 Flowchart for the extraction and preparation of scorpions and centipedes


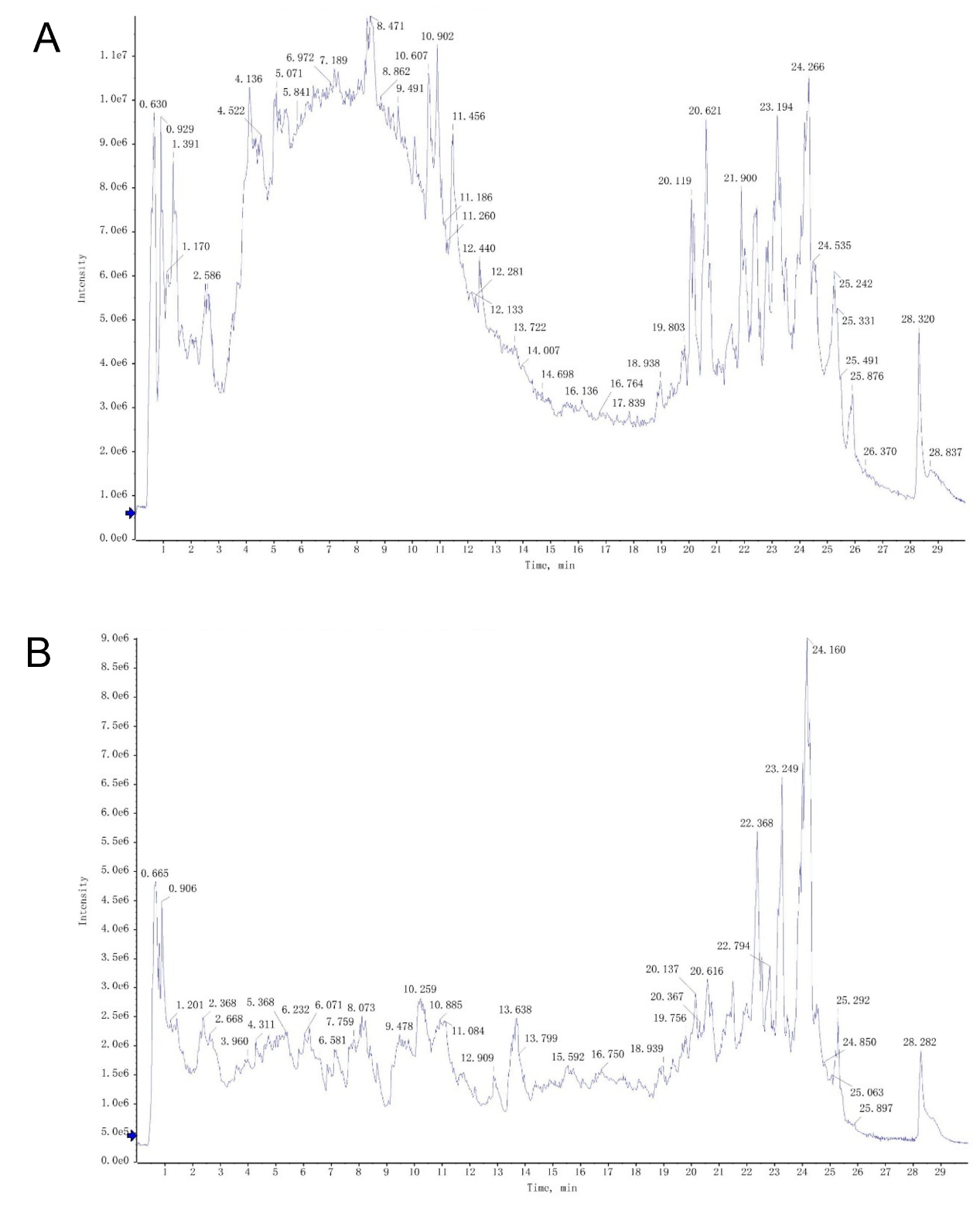


Figure S2. Component analysis of SC by LC-MS. (A)Positive ion scanning of SC. (B)negative ion scanning of SC.


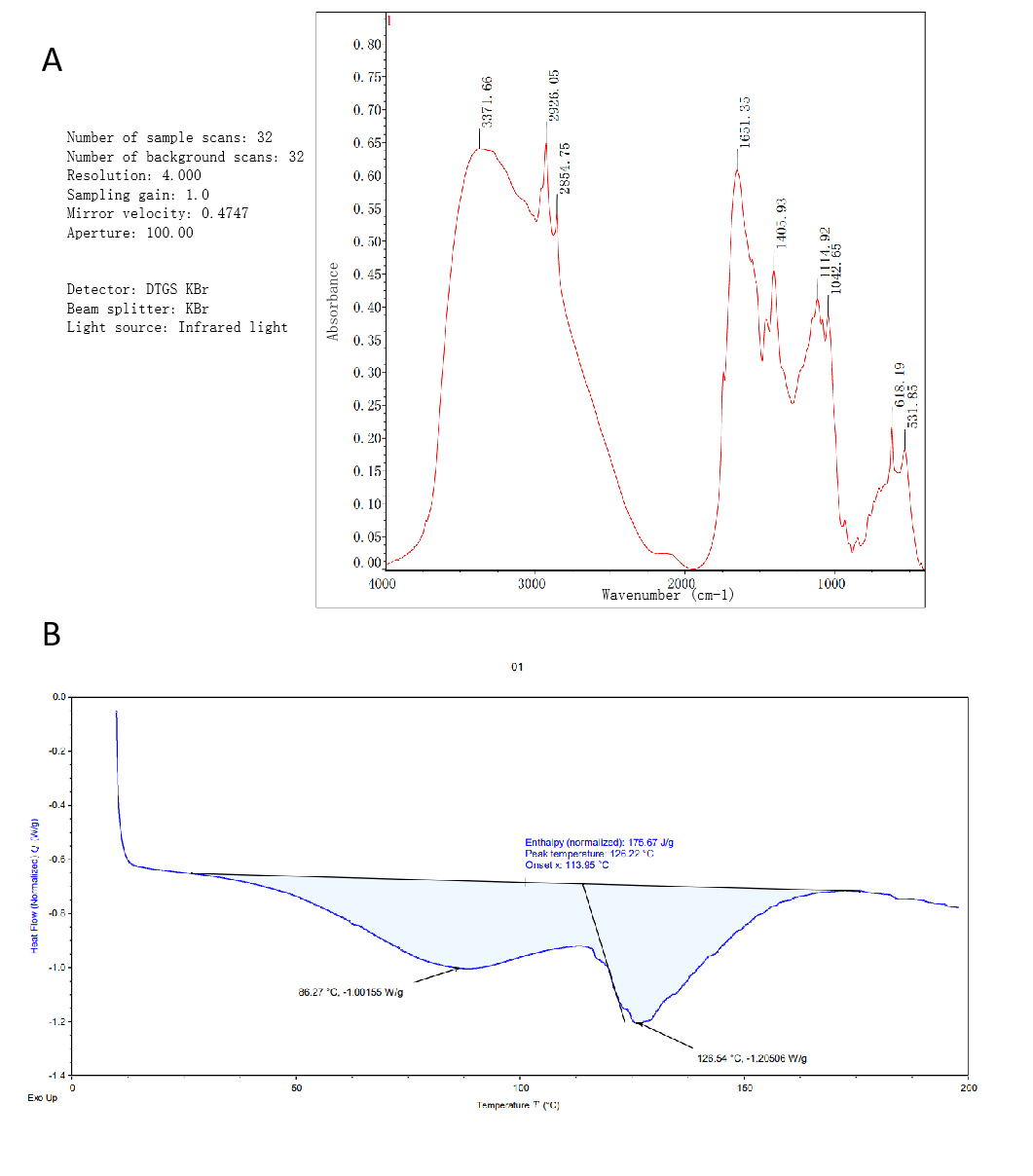


Figure S3. Fourier-Transform Infrared (FTIR) spectrum of SC, with the abscissa being wavenumber (cm⁻¹) and the ordinate being absorbance (A); Differential Scanning Calorimetry (DSC) curveof SC, with the abscissa being temperature (°C) and the ordinate being heat flow change (B).


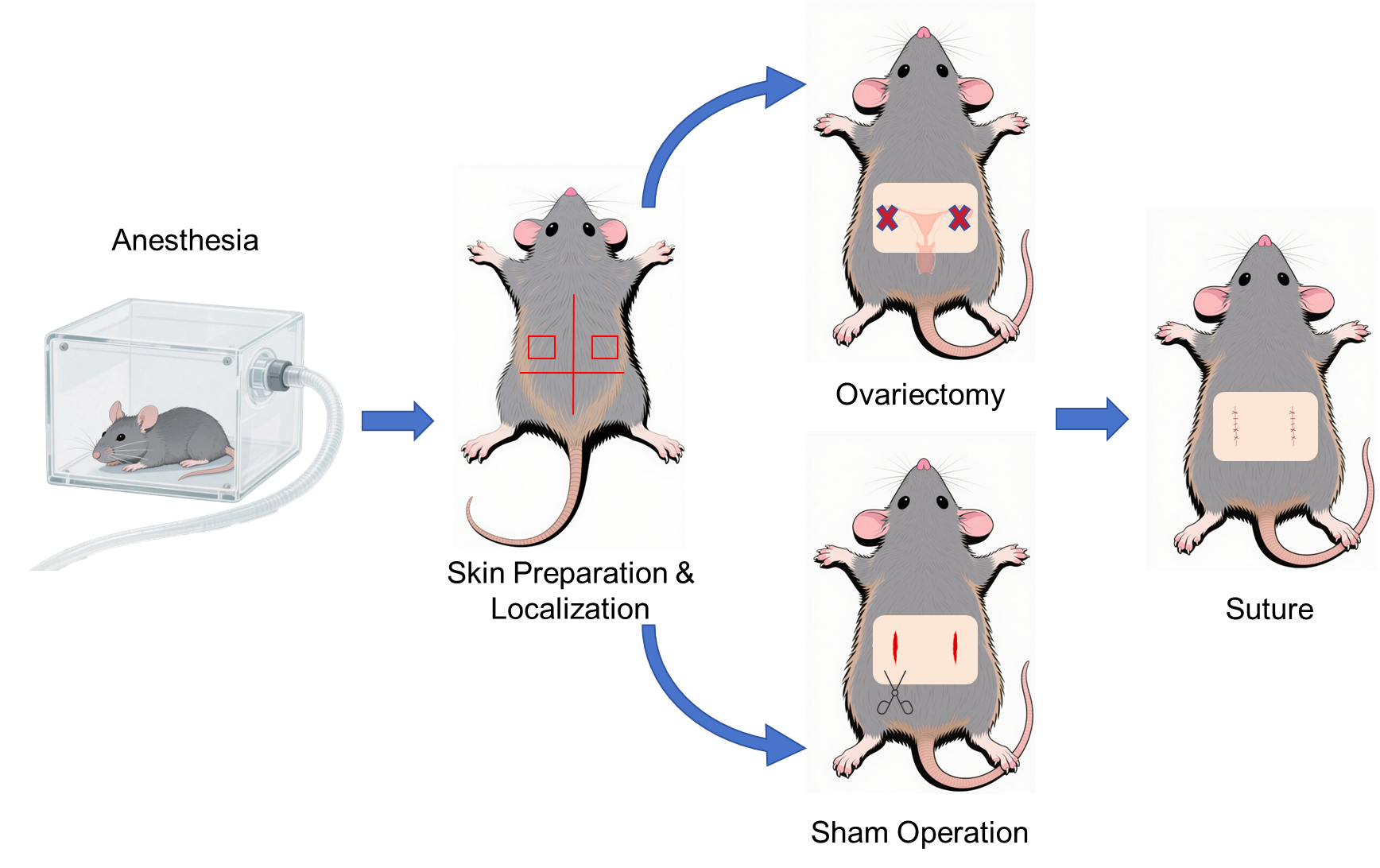


Figure S4. Bilateral ovariectomy was performed on the mice


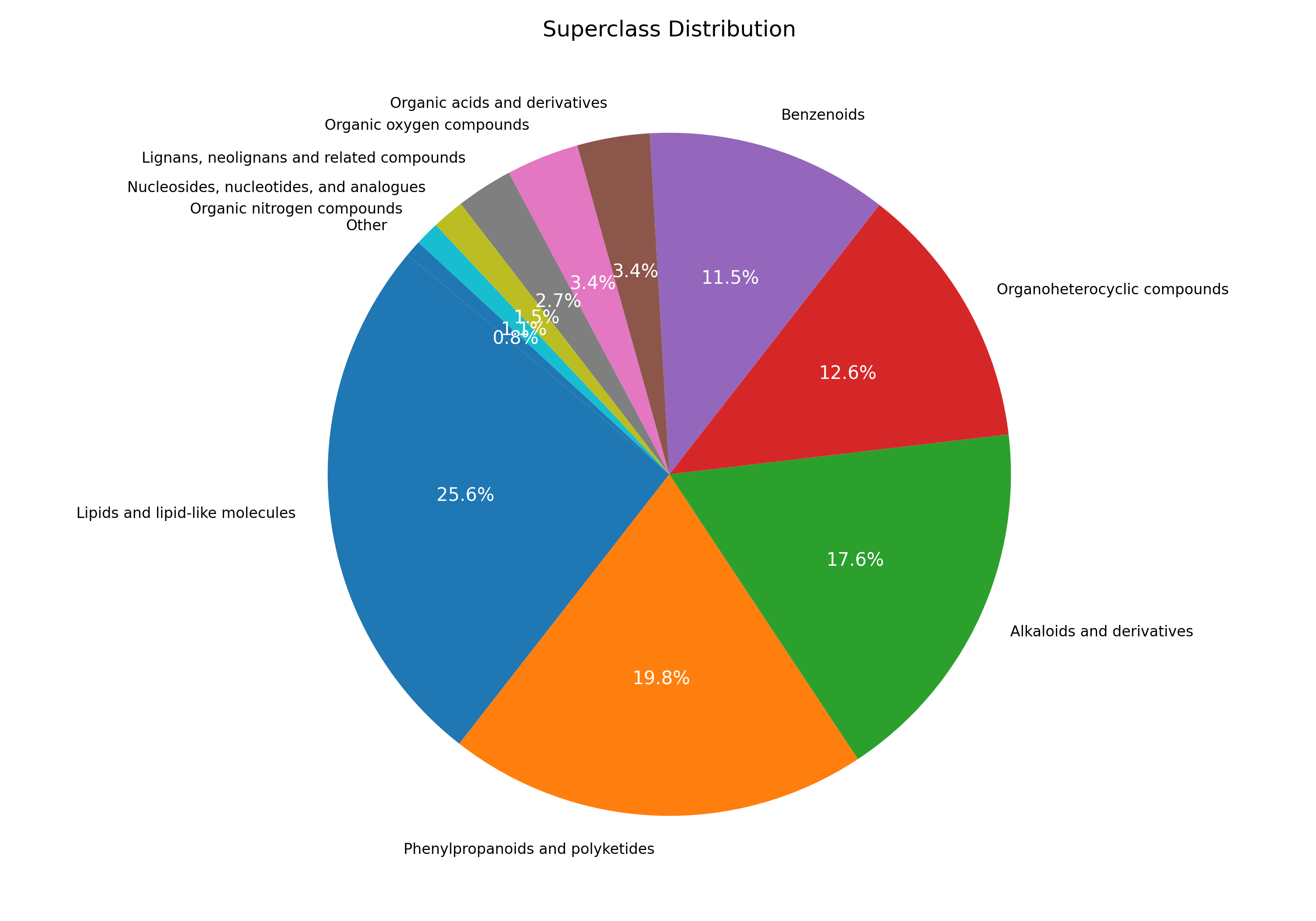

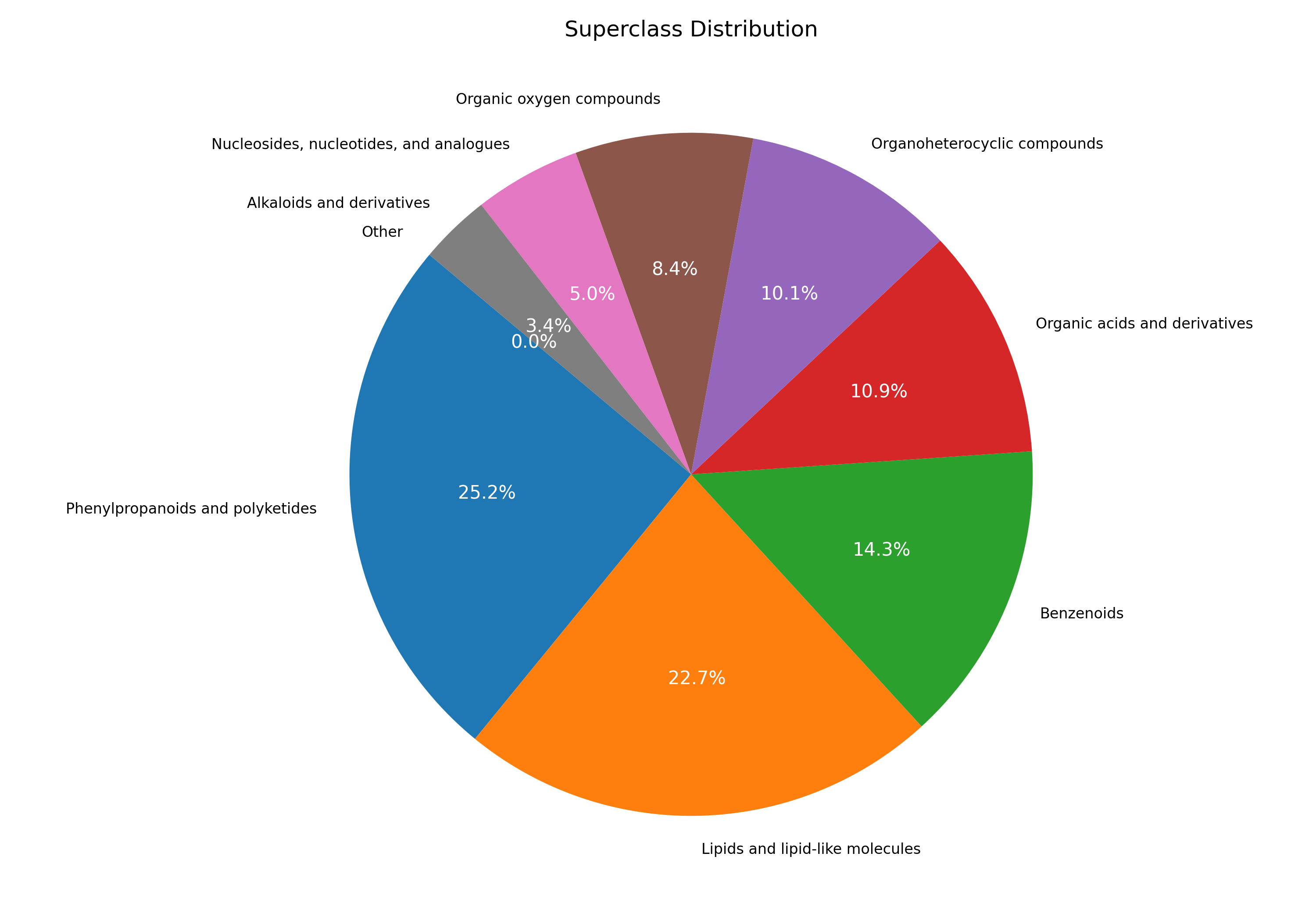


Figure S5. Superclass distribution of SC by LC-MS. (Up) Positive ion scanning of SC. (Down) negative ion scanning of SC.
